# Susceptibility Genes in Thyroid Autoimmunity

**DOI:** 10.1080/17402520400008897

**Published:** 2005-03

**Authors:** Yoshiyuki Ban, Yaron Tomer

**Affiliations:** Division of Endocrinology, Diabetes, and Bone Diseases Department of Medicine Mount Sinai School of Medicine New York NY USA

## Abstract

The autoimmune thyroid diseases (AITD) are complex diseases which are caused by an interaction between susceptibility genes and environmental triggers. Genetic susceptibility in combination with external factors (e.g. dietary iodine) is believed to initiate the autoimmune response to thyroid antigens. Abundant epidemiological data, including family and twin studies, point to a strong genetic influence on the development of AITD. Various techniques have been employed to identify the genes contributing to the etiology of AITD, including candidate gene analysis and whole genome screening. These studies have enabled the identification of several loci (genetic regions) that are linked with AITD, and in some of these loci, putative AITD susceptibility genes have been identified. Some of these genes/loci are unique to Graves' disease (GD) and Hashimoto's thyroiditis (HT) and some are common to both the diseases, indicating that there is a shared genetic susceptibility to GD and HT. The putative GD and HT susceptibility genes include both immune modifying genes (e.g. HLA, CTLA-4) and thyroid specific genes (e.g. TSHR, Tg). Most likely, these loci interact and their interactions may influence disease phenotype and severity.


*Clinical & Developmental Immunology*, March 2005; 12(1): 47–58

Taylor & Francis

Taylor & Francis Group

### Susceptibility genes in thyroid autoimmunity

YOSHIYUKI BAN, & YARON TOMER

*Division of Endocrinology, Diabetes, and Bone Diseases, Department of Medicine, Mount Sinai School of Medicine, New York, NY, USA*

**Abstract**
The autoimmune thyroid diseases (AITD) are complex diseases which are caused by an interaction between susceptibility genes and environmental triggers. Genetic susceptibility in combination with external factors (e.g. dietary iodine) is believed to initiate the autoimmune response to thyroid antigens. Abundant epidemiological data, including family and twin studies, point to a strong genetic influence on the development of AITD. Various techniques have been employed to identify the genes contributing to the etiology of AITD, including candidate gene analysis and whole genome screening. These studies have enabled the identification of several loci (genetic regions) that are linked with AITD, and in some of these loci, putative AITD susceptibility genes have been identified. Some of these genes/loci are unique to Graves' disease (GD) and Hashimoto's thyroiditis (HT) and some are common to both the diseases, indicating that there is a shared genetic susceptibility to GD and HT. The putative GD and HT susceptibility genes include both immune modifying genes (e.g. HLA, CTLA-4) and thyroid specific genes (e.g. TSHR, Tg). Most likely, these loci interact and their interactions may influence disease phenotype and severity.

**Keywords:** *Gene, thyroid, Graves' disease, Hashimoto's thyroiditis, linkage, association*

### Introduction

The autoimmune thyroid diseases (AITD) include a number of conditions which have in common cellular and humoral immune responses targeted at the thyroid gland. The AITD include Graves' disease (GD) and Hashimoto's thyroiditis (HT), both of which are characterized by infiltration of the thyroid by T and B cells reactive with thyroid antigens, production of thyroid autoantibodies, with the resultant clinical manifestations (hyperthyroidism in GD and hypothyroidism in HT) (reviewed in Weetman (1996) and Davies (2000)). There is abundant evidence for a major genetic influence on the development of AITD (reviewed in Tomer et al. (1997a) and Brix et al. (1998a)). Therefore, the current paradigm is that AITD are complex diseases in which susceptibility genes and environmental triggers act in concert to initiate the autoimmune response to the thyroid. In this review, we focus on the genes already found to contribute to AITD. While the proof that a gene causes AITD requires functional studies, the genes we will discuss are strong candidates, and their functions are now being investigated. We will not discuss in detail the genetic regions linked with AITD in which no candidate gene has yet been identified.

### Genetic epidemiology of AITD

The familial occurrence of AITD has been reported by investigators for many years. Early studies showing familial aggregation of AITD were mostly observational, based on careful family histories from patients (Bartels 1941, Martin 1945). Later, in the 1960s, Hall and Stanbury (1967) showed that 33% of siblings of patients with GD or HT developed AITD themselves. Additionally, they found that 56% of siblings of AITD patients had thyroid antibodies (TAbs) (Hall and Stanbury 1967). A recent survey by our own group revealed that 41/114 (36%) of GD patients with ophthalmopathy reported a family history of AITD and 26/114 (23%) had a first degree relative with AITD (Villanueva et al. 2000).

The sibling risk ratio ($\lambda s$), which is the ratio of the prevalence of the disease in siblings of affected individuals compared to the prevalence of the disease

Correspondence: Y. Tomer, Division of Endocrinology, Diabetes and Bone Diseases, Box 1055, Mount Sinai Medical Center, One Gustave L. Levy Place, New York, NY 10029, USA. Tel: 1 212 241 1476. Fax: 1 212 241 4218. E-mail: yaron.tomer@mssm.edu

ISSN 1740-2522 print/ISSN 1740-2530 online © 2005 Taylor & Francis Group Ltd.
DOI: 10.1080/17402520400008897

in the general population (Risch, 1990), serves as a good estimate of disease heritability, with $\lambda s > 5$ considered significant. The $\lambda s$ in AITD has been estimated to be between 5.9 (Brix et al. 1998a) and $>10$ in AITD (Vyse and Todd 1996, Villanueva et al. 2000), supporting a strong genetic influence on the development of AITD.

Several large twin studies have been reported from Denmark showing a higher concordance of GD in monozygotic (MZ) twins when compared to dizygotic (DZ) twins (Brix et al. 1998b, Brix et al. 2001). A recent GD twin study from California confirmed the Danish twin study results (Ringold et al. 2002). Twin studies in HT have also shown a higher concordance rate in MZ compared to DZ twins (Brix et al. 2000). The concordance rates for TAbs were also reported to be higher in MZ twins compared to DZ twins (Phillips et al. 2002). Thus, the twin data confirm the presence of a substantial inherited susceptibility to AITD.

### Susceptibility genes in AITD

### *Immune related genes*

*The human leukocyte antigen (HLA) gene (Table I)*. The major histocompatibility complex (MHC) region, encoding the HLA glycoproteins, consists of a complex of genes located on chromosome 6p21 (Todd et al. 1988). GD was initially found to be associated with HLA-B8 in Caucasians (Bech et al. 1977, Farid et al. 1980). Subsequently, it was found that GD was more strongly associated with HLA-DR3, which is now known to be in linkage disequilibrium with HLA-B8 (reviewed in Farid (1981)). The frequency of DR3 in GD patients was generally 40–55% and in the general population ~15–30% giving a RR for people with HLA-DR3 of up to 4.0 (Farid et al. 1979, Farid et al. 1980, Volpe 1990, Mangklabruks et al. 1991). A recent family-based study from UK using the transmission disequilibrium test (TDT) confirmed the results of the case control studies (Heward et al. 1998a). Among Caucasians, HLA-DQA1*0501 was also shown to be associated with GD (RR = 3.8) (Yanagawa et al. 1993, Barlow et al. 1996, Marga et al. 2001), but recent studies have suggested that the primary susceptibility allele in GD is indeed HLA-DR3 (HLA-DRB1*03) (Zamani et al. 2000). However, the exact amino-acid sequence in the DRβ1 chain conferring susceptibility to GD is unknown. In other autoimmune diseases including Type 1 diabetes (T1D) (Todd et al. 1987), there is persuasive evidence that the disease is associated with specific amino-acid sequences of the DRB1 and DQ genes. We recently sequenced the HLA-DRB1 locus in a population of GD patients and controls (Ban et al. 2004a). Sequence analysis showed an increased frequency of arginine at position 74 of the HLA-DRβ1 chain (DRβ-Arg-74) in GD patients compared to controls (Ban et al. 2004a). Moreover, subset analyses showed that DRβ-Arg-74 was also significantly more frequent in the HLA-DR3 negative GD patients than in controls, suggesting that the association with DRβ-Arg-74 is independent of the association with HLA-DR3. The pattern of transmission of HLA alleles from parents to offspring was also studied. A recent study suggested a preferential transmission of HLA susceptibility alleles from fathers to affected offspring, whereas maternal susceptibility alleles were not transmitted

Table I. Some HLA association studies in GD performed in Caucasians.

| Country | No. of patients | HLA allele | Relative risk/*p*-value | Reference |
|---|---|---|---|---|
| Belgium | 194 | DRB1*0301 | 2.53 | Zamani et al. (2000) |
| Canada | 175 | B8 | 3.1 | Farid et al. (1980) |
| | | DR3 | 5.7 | |
| Denmark | 86 | B8 | 2.80 | Bech et al. (1977) |
| | | Dw3 | 3.94 | |
| Germany | 253 | DR3 | 2.52 | Schleusener et al. (1989) |
| Hungary | 256 | B8 | 3.48 | Stenszky et al. (1985) |
| | | DR3 | 4.8 | |
| Sweden | 78 | B8 | 4.4 | Dahlberg et al. (1981) |
| | | DR3 | 3.9 | |
| UK | 127 | B8 | 2.77 | Kendall-Taylor et al. (1988) |
| | | DR3 | 2.13 | |
| UK | 101 | DR3 | 1.10 | Weetman et al. (1988) |
| USA | 65 | DR3 | 3.38 | Mangklabruks et al. (1991) |
| USA | 92 | DRB1*03 | 2.6 | Chen et al. (1999) |
| | | DRB1*08 | 3.2 | |
| UK | 120 | DQA1*0501 | 3.8 | Barlow et al. (1996) |
| UK | 228 | DRB1*0304 | 2.7 | Heward et al. (1998a) |
| | | DQB1*0301 | 1.9 | |
| | | DQA1*0501 | 3.2 | |
| USA | 94 | DQA1*0501 | 3.71 | Yanagawa et al. (1993) |

more frequently than expected (Segni et al. 2002). This may suggest parental imprinting in the transmission of HLA susceptibility alleles to affected offspring.

Data on HLA haplotypes in HT have been less definitive than in GD. Initial studies failed to demonstrate an association between goitrous HT and HLA A- B- or C- antigens (Irvine et al. 1978). Later studies showed an association of goitrous HT with HLA- DR5 (RR = 3.1) (Farid et al. 1981) and of atrophic HT with DR3 (RR = 5.1) (Moens et al. 1978). The association of HT with HLA-DR4 in Caucasians has been confirmed in subsequent studies (Tandon et al. 1991, Ban et al. 2002a), and further supported by studies of transgenic mice (Kong et al. 1996). An association between HT and HLA-DQw7 (DQB1*0301) has also been reported in Caucasians (Badenhoop et al. 1990, Wu et al. 1994).

Linkage studies of HLA in AITD have been largely negative (Bode et al. 1973, Roman et al. 1992, Barbesino et al. 1998). Only one recent study from UK showed weak evidence for linkage between GD and the HLA region (Vaidya et al. 1999a), and an additional study reported linkage only when conditioning on DR3 (Shields et al. 1994). It is difficult to explain why the HLA genes show consistent association with GD but no evidence for linkage. The most likely explanation is that HLA is a modulating gene for AITD but not a primary susceptibility gene.

*The cytotoxic T lymphocyte antigen-4 (CTLA-4) immune regulatory cluster on chromosome 2q33 (Table II).* Co-stimulatory molecules are critical to the activation of T cells by antigen presenting cells (APCs). APCs activate T cells by presenting to the T cell receptor an antigenic peptide bound to an HLA class II protein on the cell surface. However, a second signal is also required for T cell activation and these co-stimulatory signals may be provided by the APCs themselves or other local cells (Reiser and Stadecker, 1996). The co-stimulatory signals are provided by a variety of proteins which are expressed on APCs (e.g. B7-1, B7-2, B7h, CD-40) and interact with receptors (CD28, CTLA-4, and CD-40L) on the surface of CD4+ T-lymphocytes during antigen presentation (Reiser and Stadecker, 1996). Whereas, the binding of B7 to CD28 on T cells co-stimulates T cell activation, the presence of CTLA-4, which has a higher affinity for B7, down regulates T-cell activation by competing for the binding of B7 to CD28. A new member of this family of co-stimulatory molecules, inducible co-stimulator (ICOS) was identified by Hutloff et al. (1999). Unlike the constitutively expressed CD28, ICOS is induced on the T-cell surface and does not upregulate the production of interleukin (IL)-2, but induces the synthesis of IL-4 (Coyle et al. 2000). Interestingly, CD28, CTLA-4 and ICOS form a gene cluster in a 300 kb region on chromosome 2q33. Thus, associations of autoimmune diseases with this region may represent the effects of any of these three genes alone or in combination due to linkage disequilibrium.

Recently, there have been several reports demonstrating an association between CTLA-4 gene polymorphisms and AITDs, both GD and HT (Yanagawa et al. 1995, Nistico et al. 1996, Donner et al. 1997a,b, Kotsa et al. 1997a, Marron et al. 1997, Yanagawa et al. 1997, Braun et al. 1998, Heward et al. 1998b, 1999a, Petrone et al. 2001, Tomer 2001, Kouki et al. 2002, Nithiyananthan et al. 2002).

Since CTLA-4 is a non specific co-stimulatory molecule, it is expected to confer susceptibility to AITD and autoimmunity in general, and not

Table II. Some CTLA-4 association studies in autoimmune thyroid diseases in Caucasians and non-Caucasian population.

| CTLA-4 polymorphism | Country | Ethnic group | Dis. | No. | RR*/*P* value | Reference |
|---|---|---|---|---|---|---|
| CTLA-4(AT) | USA | Caucasians | GD | 133 | 2.82 | Yanagawa et al. (1995) |
| CTLA-4(AT) | UK | Caucasians | GD | 112 | 2.1 | Kotsa et al. (1997a) |
| | | | HT | 44 | 2.2 | |
| CTLA-4(AT) | Hong-Kong | Chinese | GD | 94 | $p = 0.037$ | Nistico et al. (1996) |
| CTLA-4(AT) | Japan | Japanese | GD + HT | 349 | 1.8 | Volpe (1990) |
| Thr/Ala $(A/G)_{49}$ | Germany | Caucasians | GD | 305 | 2.0 | Donner et al. (1997a) |
| Thr/Ala $(A/G)_{49}$ | UK | Caucasians | GD | 94 | $p = 0.003$ | Vaidya et al. (1999b) |
| Thr/Ala $(A/G)_{49}$ | UK | Caucasians | GD | 379 | 1.6 | Heward et al. (1999a) |
| Thr/Ala $(A/G)_{49}$ | UK | Caucasians | GD | 484 | $p < 0.0001$ | Allahabadia et al. (2001) |
| Thr/Ala $(A/G)_{49}$ | USA | Caucasians | GD | 85 | 1.6 | Villanueva et al. (2000) |
| Thr/Ala $(A/G)_{49}$ | Germany | Caucasians | HT | 73 | $p < 0.04$ | Donner et al. (1997b) |
| Thr/Ala $(A/G)_{49}$ | Italy | Caucasians | HT | 126 | NS* | Petrone et al. (2001) |
| Thr/Ala $(A/G)_{49}$ | UK | Caucasians | HT | 158 | 1.57 | Nithiyananthan et al. (2002) |
| Thr/Ala $(A/G)_{49}$ | Slovenia | Caucasians | TAb's | 67 | $p < 0.005$ | Zaletel et al. (2002) |
| Thr/Ala $(A/G)_{49}$ | Japan | Japanese | GD | 153 | 2.64 | Yanagawa et al. (1997) |
| Thr/Ala $(A/G)_{49}$ | Korea | Korean | GD | 97 | 1.6 | Park et al. (2000) |
| | | | HT | 110 | NS | |

*RR, relative risk; NS, not significant.

specifically to GD (Tomer 2001). Indeed, CTLA-4 was reported to be associated and linked with all forms of AITD (GD, HT, and TAbs, see below), and with many autoimmune diseases such as Type 1 diabetes (T1D) (Nistico et al. 1996, Donner et al. 1997a, Marron et al. 1997, Ueda et al. 2003), Addison's disease (Vaidya et al. 2000), and myasthenia gravis (Huang et al. 1998).

Two studies have now shown that CTLA-4 confers susceptibility to the production of TAbs. Our group has shown strong evidence for linkage between the CTLA-4 gene region and the production of TAbs with a maximum LOD score (MLS) of 4.2 (Tomer et al. 2001). Recently, another report has described an association between the G allele of the CTLA-4 A/$G_{49}$ SNP and thyroid autoantibody diathesis (Zaletel et al. 2002). Since the development of TAbs often represents the pre-clinical stage of AITD (Vanderpump et al. 1995), it is possible that CTLA-4 predisposes non-specifically to the development of thyroid autoimmunity. Additional genetic and/or environmental factors must be necessary for the development of the specific GD/HT phenotypes (Tomer 2001).

As mentioned, the region on chromosome 2q33 containing the CTLA-4 gene harbors in addition the CD28 and ICOS genes and it was unclear whether the CTLA-4 gene itself or another immune regulatory gene in the region was involved in the genetic susceptibility to AITD. Recently, we tested additional genes and markers in the 2q33 region, and the strongest association was with the CTLA-4 markers (Tomer et al. 2001). These results were in agreement with results obtained in T1DM (Marron et al. 2000, Wood et al. 2002). More recently, Ueda et al. (2003) also showed that CTLA-4 was indeed the AITD susceptibility gene in this region. They identified a new 3′ untranslated region SNP that was strongly associated with AITD.

*The CD40 gene*. Two linkage studies, one by our group (Tomer et al. 1998) and one by Pearce et al. (1999) have shown evidence that a locus on 20q11 was linked with GD. This GD locus was not linked to HT, since analysis of the data for the HT families gave strongly negative LOD scores. Moreover, in families with GD- and HT-affected individuals, the locus was linked only with GD, demonstrating its high specificity for GD (Tomer et al. 1998, Wood et al. 2002). The CD40 gene, an important regulator of B cell function, is located within the linked region on chromosome 20q11 and, therefore, it was a likely positional candidate gene for GD. CD40 is a transmembrane glycoprotein that is expressed predominantly on B cells, and also on monocytes, dendritic cells, epithelial cells and other cells (reviewed in Durie et al. (1994)). It is a member of the tumor necrosis factor receptor superfamily and it binds to a ligand (CD40L or CD154), which is expressed mainly on activated T cells. Binding of CD40L to CD40 induces B cells to proliferate and undergo immunoglobulin isotype switching (Banchereau et al. 1994). CD40 has been shown to play an important role in the regulation of humoral immunity, central and peripheral T-cell tolerance, and APC function (reviewed in Foy et al. (1996)). Moreover, *in vivo* blockade of CD40 has been shown to suppress the induction of experimental autoimmune thyroiditis (Carayanniotis et al. 1997). Therefore, we tested whether CD40 was the GD susceptibility gene on chromosome 20q11. Sequencing of the CD40 gene revealed a C/T SNP in the 5′ untranslated region (5′ UTR) of the gene. Analysis of the CD40 5′ UTR SNP in 154 Caucasian GD patients and 118 Caucasian controls showed an association between the CC genotype and GD but with a low relative risk of 1.6 (Tomer et al. 2002a). TDT analysis also showed preferential transmission of the C allele of the CD40 5′ UTR SNP to affected individuals (Tomer et al. 2002a). Other investigators who found evidence for linkage in this region have not found an association between this SNP and GD in their dataset (Pearce, personal communication) and it is possible that other polymorphisms in the CD40 gene, or another gene in linkage disequilibrium with CD40, is the GD susceptibility gene.

*Other immune related genes*. Other immune related genes tested for association with GD include the T cell receptor β chain (Demaine et al. 1987, Weetman et al. 1987, Mangklabruks et al. 1991), the IgG heavy chain (IgH) gene (Roman et al. 1989, Fakhfakh et al. 1999), the IL-1 receptor antagonist gene (Blakemore et al. 1995, Cuddihy and Bahn 1996, Muhlberg et al. 1998, Heward et al. 1999b), tumor necrosis factor α (TNFα) gene (Barbesino et al. 1998), interferon γ gene (Siegmund et al. 1998), the transporters associated with antigen presentation (TAP) genes (Rau et al. 1997), and the IL-4 gene (Heward et al. 2001). However, none of these have produced replicable associations with GD. The vitamin D binding protein, which may have some immune modulatory functions, has also been reported to be associated with GD (Pani et al. 2002).

### Thyroid associated genes

### *The thyroglobulin (Tg) gene*

Two studies have found evidence for linkage between a locus on chromosome 8q24 and AITD. Our group has shown strong evidence for linkage at the Tg gene locus with an MLS of 3.5 between D8S514 and D8S284 (Tomer et al. 2002b). Recently, another study in Japanese sib-pairs identified a major AITD locus on 8q24 very close to the locus, which we identified

(Sakai et al. 2001). Since the Tg gene was located within this linked region we proceeded to analyze the Tg gene directly. We identified two new Tg microsatellites in intron 10 (designated Tgms1) and intron 27 (designated Tgms2). Linkage analysis using Tgms2 gave a 2-point LOD score of 2.1 and a multipoint LOD score of 2.9, confirming that it was the Tg gene linked with AITD (Tomer et al. 2002b).

We then used the same two Tg microsatellites to test whether the Tg gene was associated as well as linked with AITD. Using an unselected group of 190 Caucasian GD patients and 134 age- and sex-matched Caucasian controls we found only a weak association between Tgms2 and AITD ($p = 0.05$, RR = 1.4) (Tomer et al. 2002b). However, the association was more impressive when the probands from the linked families ($n = 32$) were used ($p = 0.004$, RR = 2.3). TDT analysis also showed an association of Tgms2 with AITD ($p = 0.02$, Table III), but with a different allele, suggesting that Tgms2 was in linkage disequilibrium with another polymorphism of the Tg gene. These results have been replicated recently in a UK dataset (Collins et al. 2003). As in our study, the UK study also showed a significant association between Tgms2 and AITD ($p < 0.001$). Moreover, the same Tgms2 allele that we found to be associated with AITD was found to be associated by Collins et al. (2003). Thus, the Tg gene was both linked and associated with AITD and, therefore, is an important AITD susceptibility gene. Recently, sequence changes in the Tg gene which were associated with AITD were identified (Ban et al. 2003). Case control association studies for 14 Tg SNPs in AITD patients and controls showed that one SNP cluster (in exons 10–12) and an exon 33 SNP were significantly associated with AITD (Ban et al. 2003). However, it remains possible that another gene on 8q24 in linkage disequilibrium with Tg was the AITD susceptibility gene responsible for the observed linkage and association at this locus.

Table III. Transmission disequilibrium test for markers D8S284, Tgms1 and Tgms2 in 102 AITD families.

| Marker | Allele/ Haplotype | Transmitted | Untransmitted | *p*-value |
|---|---|---|---|---|
| D8S284 | 3 | 54 | 34 | **0.03** |
| | 9 | 6 | 16 | **0.03** |
| | All others | 111 | 121 | NS* |
| Tgms2 | 3 | 48 | 34 | NS |
| | 4 | 14 | 4 | **0.02** |
| | 7 | 32 | 52 | **0.02** |
| | All others | 62 | 66 | NS |
| D8S284/ Tgms1 | 3/3 | 32 | 12 | **0.002** |
| | All others | 101 | 121 | NS |

*NS, not significant; Tgms1, microsatellite marker located in intron 10 of the Tg gene; Tgms2, microsatellite marker located in intron 27 of the Tg gene.

### *The TSH receptor (TSHR) gene*

The hallmark of GD is the production of the TSHR antibodies. Therefore, the TSHR gene was thought to be a likely candidate gene for GD. Three common germline SNPs of the TSHR have been described (Tonacchera and Pinchera 2000). Two of them are located in the extracellular domain of the TSHR: an aspartic acid to histidine substitution at position 36 (D36H), and a proline to threonine substitution at position 52 (P52T). The third SNP is a substitution of glutamic acid for aspartic acid (D727E) within the intracellular domain of the receptor. Most studies on the contribution of the TSHR gene to the genetic susceptibility to GD have focused on the two SNPs in the extracellular domain of the TSHR (Cuddihy et al. 1995, Kotsa et al. 1997b, Allahabadia et al. 1998, Simanainen et al. 1999, Chistyakov et al. 2000, Kaczur et al. 2000), because this domain is the major site for TSH and TSHR antibody binding. Amino acid changes in the extracellular domain of the TSHR could theoretically change the amino acid sequence of TSHR T-cell epitopes (Rapoport et al. 1998). Initial studies suggested that the P52T SNP was associated with GD in females (Cuddihy et al. 1995). However, other authors were unable to confirm the association between the P52T SNP and GD in Caucasians (Kotsa et al. 1997b, Allahabadia et al. 1998, Simanainen et al. 1999, Chistyakov et al. 2000, Kaczur et al. 2000). The D36H SNP has also been reported not to be associated with GD (Simanainen et al. 1999). Linkage studies in GD families using three microsatellite markers within introns 2 and 7 of the TSHR gene were also negative in Caucasians (De Roux et al. 1996, Tomer et al. 1999). Recently, the D727E SNP was reported to be associated with GD in a Caucasian Russian population (Chistiakov et al. 2002), but these results were not replicated in a subsequent study (Muhlberg et al. 2000). Recently, we also tested whether the D727E SNP was associated with GD, but there was no association between the D727E SNP and GD, and no effect of the D727E SNP on the GD phenotype or disease severity was seen (Ban et al. 2002b). In addition, the frequency of the G allele was not increased in patients with more severe forms of GD (i.e. ophthalmopathy and goiter) and in patients with early disease onset. Our own study and other negative TSHR studies have not excluded a weak association between GD and the TSHR gene since very large datasets may be needed to detect associations with low RRs. We, therefore, performed a meta-analysis combining our data with the data reported in the previous two negative TSHR studies. The results showed a weak association between the D727E SNP E allele and GD ($p = 0.03$, RR = 1.6) (Ban et al. 2002b). Therefore, at this time it remains possible that the TSHR is a minor susceptibility gene for GD.

*The thyroid peroxidase (TPO) gene*

The TPO gene was tested for linkage and association with AITD in two studies using a microsatellite inside the TPO gene. However, these studies showed no evidence of linkage and/or association of the TPO gene with AITD (Pirro et al. 1995, Tomer et al. 1997b). Therefore, the TPO gene is most likely not a major susceptibility gene for AITD.

### The effect of ethnicity in the development of AITD

*The HLA gene (Table IV)*

As previously mentioned, HLA-DR3 is associated with GD in Caucasians. The HLA genes were also shown to be associated with GD in non-Caucasians, albeit the associated alleles were different (Table IV). Studies in the Japanese population have shown associations of GD with HLA-B35 (Kawa et al. 1977, Inoue et al. 1992). However, other class I and II HLA alleles have also been reported to be increased in Japanese GD patients (Katsuren et al. 1994, Onuma et al. 1994, Ohtsuka and Nakamura 1998). In Chinese an increased frequency of HLA-Bw46 has been reported (Chan et al. 1978, Tan et al. 1988, Yeo et al. 1989, Chan et al. 1993, Cavan et al. 1994). It is interesting that in Asians the HLA associations are with class I genes while in Caucasians they are with class II genes. This may imply that other non-HLA genes in the region in linkage disequilibrium with class I genes are the susceptibility genes in Asians. In contrast, DR3 is believed to be the causative gene in Caucasians (see below). In African–Americans an increased frequency of HLA DRB3*0202 has been reported (Table IV) (Chen et al. 2000a). Interestingly, one study of a mixed population in Brazil showed association with HLA-DR3 implying that this allele may confer susceptibility in other ethnic groups and not just Caucasians (Maciel et al. 2001). Alternatively, this Brazilian population may have been mostly of European ancestry.

Also, HLA association studies in HT have not been consistent in non-Caucasian ethnic groups, e.g. HLA-DRw53 in Japanese (Honda et al. 1989), and HLA-DR9 in Chinese (Hawkins et al. 1987) (Table IV). In addition, linkage studies of HLA in AITD have been consistently negative in non-Caucasians, including Chinese (Hawkins et al. 1985a) and Japanese (Sakai et al. 2001). The negative linkage studies imply that HLA are also minor AITD genes in non-Caucasians.

*The CTLA-4 gene*

The association between GD and the CTLA-4 3′ UTR microsatellite and $A/G_{49}$ SNP has been consistent across populations of different ethnic backgrounds such as Japanese (Yanagawa et al. 1997, Akamizu et al. 2000), and Koreans (Park et al. 2000). As was reported in Caucasians (Heward et al. 1998b), the $C/T_{-318}$ SNP of CTLA-4 has not been associated with GD in Chinese (Heward et al. 1998b). It has also been reported that the frequency of the G allele and the GG genotype of the CTLA-4 $A/G_{49}$ SNP was significantly higher in GD patients who did not go into remission after five years on anti-thyroid medications in Japanese (Kinjo et al. 2002). Similarly, CTLA-4 has been reported to be associated with HT in non-Caucasians including Japanese (Akamizu et al. 2000, Sale et al. 1997).

Table IV. Some HLA association studies in AITD performed in non-Caucasian populations.

| Country | Ethnic group | Dis. | No. of Patients | HLA allele(s) | RR*/p-value | Reference |
|---|---|---|---|---|---|---|
| Hong Kong | Chinese | GD | 132 | Bw46 | 4.8 | Hawkins et al. (1985b) |
| Hong Kong | Chinese | GD | 97 | B46 | 2.3 | Cavan et al. (1994) |
| | | | | DR9 | 2.2 | |
| | | | | DQB1*0303 | 3.2 | |
| Singapore | Chinese | GD | 35 | B46 | 8.2 | Chan et al. (1993) |
| Singapore | Chinese | GD | 159 | Bw46 | 4.2 | Yeo et al. (1989) |
| Japan | Japanese | GD | 33 | Bw35 | $p < 0.02$ | Kawa et al. (1977) |
| Japan | Japanese | GD | 106 | B46 | $p < 0.000$ | Onuma et al. (1994) |
| Japan | Japanese | GD | 76 | A2 | 2.86 | Dong et al. (1992) |
| | | | | DPB1*0501 | 5.32 | |
| Korea | Korean | GD | 128 | B13 | 3.8 | Cho et al. (1987) |
| | | | | DR5 | 4.4 | |
| | | | | DRw8 | 2.3 | |
| India | Asian Indian | GD | 57 | B8 | 4.1 | Tandon et al. (1990) |
| | | | | DQw2 | 5.4 | |
| USA | African–American | GD | 73 | No association | | Sridama et al. (1987) |
| USA | African–American | GD | 49 | DRB3*0202 | 3.6 | Chen et al. (2000b) |
| South Africa | African | GD | 103 | DR1 | 3.5 | Omar et al. (1990) |
| | | | | DR3 | 2.4 | |
| China | Chinese | HT | 53 | DRw9 | $p < 0.05$ | Hawkins et al. (1987) |
| Japan | Japanese | HT | 99 | DRw53 | 3.33 | Honda et al. (1989) |

* RR, relative risk.

Although no linkage study has been reported in non-Caucasians, the association of CTLA-4 across populations of different ethnic backgrounds shows that it is an important susceptibility gene for thyroid autoimmunity.

*Other immune related genes*

The IgH gene was found to be associated with GD in the Japanese (Nakao et al. 1980, Nagataki, 1986). However, these results have not been reproduced in Caucasians (Roman et al. 1989, Fakhfakh et al. 1999). This might imply that the IgH gene may contribute to the susceptibility to GD only in the Japanese if a founder effect exists. Alternatively, these could result from random event due to sampling small populations. Recently, the TNF $\alpha$ gene (Kamizono et al. 2000) and the vitamin D receptor, which may have some immune modulatory functions, has been reported to be associated with GD in Japanese (Ban et al. 2000). A C/T SNP in the promoter region of the CD40 gene has also been reported to be associated with GD in Koreans (Kim et al. 2003). These results need to be confirmed and it cannot be excluded that other genes in linkage disequilibrium with these genes are the susceptibility genes at these loci. Other immune related genes such as the interferon $\gamma$ gene have not been tested yet in non-Caucasians, and warrant further studies.

*The Tg gene*

A Japanese whole genome screen in 123 Japanese sib-pair families identified two loci giving strong evidence for linkage [i.e. MLS $> 2.0$]. One of these loci is located on chromosome 8q24 and showed evidence for linkage with both AITD (MLS $= 2.31$) and HT (MLS $= 3.77$) (Sakai et al. 2001). This locus is identical to the one found to be linked in Caucasians (Tomer et al. 2002b) and contains the Tg gene. Since the Tg locus was linked with AITD both in Caucasians and in Japanese, this supports that it is a major gene. Indeed, Tgms2 was associated with HT in Japanese ($p = 0.04$) (Ban et al. 2004b). However, since the significance was borderline, this association could still be due to random chance event.

*The TSHR gene*

Associations between AITD and TSHR microsatellite markers have been reported in the Japanese (Sale et al. 1997, Akamizu et al. 2000). However, these results have not been reproduced in Caucasians (Kotsa et al. 1997b, Allahabadia et al. 1998, Simanainen et al. 1999, Chistyakov et al. 2000, Kaczur et al. 2000, Villanueva et al. 2000). These results suggest that TSHR gene may contribute to the susceptibility to GD only in Japanese especially if there is a founder effect. For example, NOD2 mutations in Crohn's disease were shown only in Caucasians, and not in Japanese (Yamazaki et al. 2002).

### Conclusion

The AITD are complex diseases believed to be caused by the combined effects of multiple susceptibility genes and environmental triggers. There are sufficient epidemiologic data to support an important genetic contribution to the development of AITD, and in the past few years several loci and genes have shown evidence for linkage and/or association with AITD. The genetic susceptibility to AITD seems to involve several genes with varying effects. With the completion of the human genome project and the establishment of large SNP databases the identification of additional AITD susceptibility genes will become more feasible.

The AITD loci identified so far show that some putative AITD susceptibility genes may be immune related genes which increase the susceptibility to autoimmunity in general (e.g. HLA, CTLA-4) while others may be specific to AITD (e.g. TSHR, Tg). The next step in investigating the role of these genes in the development of AITD is by functional studies and genotype–phenotype correlations. Preliminary functional studies have been performed for HLA (Sawai and DeGroot 2000) and CTLA-4 (Kouki et al. 2000, Xu et al. 2002). More functional studies are needed for these and other genes which have shown association with AITD.

It is most likely that the susceptibility genes for AITD interact and that their interactions may influence disease phenotype and severity (Tomer et al. 1999). The molecular basis for the interactions between susceptibility genes in complex diseases is unknown. These interactions could represent the cumulative effect of increased statistical risk, or alternatively, there may be molecular interactions between the susceptibility genes or their products which ultimately determine disease phenotype. We are slowly progressing towards identification of the AITD susceptibility genes and once they are identified, we will begin to understand the underlying molecular mechanisms by which they induce thyroid autoimmunity.

### Acknowledgements

We thank Drs Terry F Davies and David A Greenberg for their teaching, support and ever ready help in our joint studies. This work was supported in part by grants DK61659 and DK58072 from NIDDKD (to YT).

### References

Akamizu T, Sale MM, Rich SS, Hiratani H, Noh JY, Kanamoto N, Saijo M, Miyamoto Y, Saito Y, Nakao K, Bowden DW. 2000.

Association of autoimmune thyroid disease with microsatellite markers for the thyrotropin receptor gene and CTLA-4 in Japanese patients. Thyroid 10:851–858.

Allahabadia A, Heward JM, Mijovic C, Carr-Smith J, Daykin J, Cockram C, Barnett AH, Sheppard MC, Franklyn JA, Gough SC. 1998. Lack of association between polymorphism of the thyrotropin receptor gene and Graves' disease in United Kingdom and Hong Kong Chinese patients: Case control and family-based studies. Thyroid 8:777–780.

Allahabadia A, Heward JM, Nithiyananthan R, Gibson SM, Reuser TT, Dodson PM, Franklyn JA, Gough SC. 2001. MHC class II region, CTLA4 gene, and ophthalmopathy in patients with Graves' disease. Lancet 358:984–985.

Badenhoop K, Schwartz G, Walfish PG, Drummond V, Usadel KH, Bottazzo GF. 1990. Susceptibility to thyroid autoimmune disease: molecular analysis of HLA-D region genes identifies new markers for goitrous Hashimoto's thyroiditis. J Clin Endocrinol Metab 71:1131–1137.

Ban Y, Taniyama M, Ban Y. 2000. Vitamin D receptor gene polymorphism is associated with Graves' disease in the Japanese population. J Clin Endocrinol Metab 85:4639–4643.

Ban Y, Davies TF, Greenberg DA, Concepcion ES, Tomer Y. 2002a. The influence of human leucocyte antigen (HLA) genes on autoimmune thyroid disease (AITD): results of studies in HLA-DR3 positive AITD families. Clin Endocrinol (Oxf) 57:81–88.

Ban Y, Greenberg DA, Concepcion ES, Tomer Y. 2002b. A germline single nucleotide polymorphism at the intracellular domain of the human thyrotropin receptor does not have a major effect on the development of Graves' disease. Thyroid 12:1079–1083.

Ban Y, Greenberg DA, Concepcion ES, Skrabanek L, Villanueva R, Tomer Y. 2003. Amino acid substitutions in the thyroglobulin gene are associated with susceptibility to human and murine autoimmune thyroid disease. Proc Natl Acad Sci USA 100:15119–15124.

Ban Y, Davies TF, Greenberg DA, Concepcion ES, Osman R, Oashi T, Tomer Y. 2004a. Arginine at position 74 of the HLA-DRβ1 chain is associated with Graves' disease. Genes Immun 5:203–208.

Ban Y, Tozaki T, Taniyama M, Tomita M, Ban Y. 2004b. Association of a thyroglobulin gene polymorphism with Hashimoto's thyroiditis in the Japanese population. Clin Endocrinol (Oxf), (in press).

Banchereau J, Bazan F, Blanchard D, Briere F, Galizzi JP, van Kooten C, Liu YJ, Rousset F, Saeland S. 1994. The CD40 antigen and its ligand. Annu Rev Immunol 12:881–922.

Barbesino G, Tomer Y, Concepcion ES, Davies TF, Greenberg DA. 1998. Linkage analysis of candidate genes in autoimmune thyroid disease: I. Selected immunoregulatory genes. J Clin Endocrinol Metab 83:1580–1584.

Barlow ABT, Wheatcroft N, Watson P, Weetman AP. 1996. Association of HLA-DQA1*0501 with Graves' disease in English Caucasian men and women. Clin Endocrinol 44:73–77.

Bartels ED. 1941. Twin examinations: Heredity in Graves' disease. Copenhagen: Munksgaad :32–36.

Bech K, Lumholtz B, Nerup J, Thomsen M, Platz P, Ryder LP, Svejgaard A, Siersbaek-Nielsen K, Hansen JM, Larse JH. 1977. HLA antigens in Graves' disease. Acta Endocrinol 86:510–516.

Blakemore AIF, Watson PF, Weetman AP, Duff GW. 1995. Association of Graves' disease with an allele of the interleukin-1 receptor antagonist gene. J Clin Endocrinol Metab 80:111–115.

Bode HH, Dorf ME, Forbes AP. 1973. Familial lymphocytic thyroiditis: analysis of linkage with histocompatibility and blood group. J Clin Endocrinol Metab 37:692–697.

Braun J, Donner H, Siegmund T, Walfish PG, Usadel KH, Badenhoop K. 1998. CTLA-4 promoter variants in patients with Graves' disease and Hashimoto's thyroiditis. Tissue Antigens 51:563–566.

Brix TH, Kyvik KO, Hegedus L. 1998a. What is the evidence of genetic factors in the etiology of Graves' disease? A brief review. Thyroid 8:727–734.

Brix TH, Christensen K, Holm NV, Harvald B, Hegedus L. 1998b. A population-based study of Graves' diseases in Danish twins. Clin Endocrinol 48:397–400.

Brix TH, Kyvik KO, Hegedus L. 2000. A population-based study of chronic autoimmune hypothyroidism in Danish twins. J Clin Endocrinol Metab 85:536–539.

Brix TH, Kyvik KO, Christensen K, Hegedus L. 2001. Evidence for a major role of heredity in Graves' disease: A population-based study of two Danish twin cohorts. J Clin Endocrinol Metab 86:930–934.

Carayanniotis G, Masters SR, Noelle RJ. 1997. Suppression of murine thyroiditis via blockade of the CD40–CD40L interaction. Immunology 90:421–426.

Cavan DA, Penny MA, Jacobs KH, Kelly MA, Jenkins D, Mijovic C, Chow C, Cockram CS, Hawkins BR, Barnett AH. 1994. The HLA association with Graves' disease is sex-specific in Hong Kong Chinese subjects. Clin Endocrinol (Oxf) 40:63–66.

Chan SH, Yeo PP, Lui KF, Wee GB, Woo KT, Lim P, Cheah JS. 1978. HLA and thyrotoxicosis (Graves' disease) in Chinese. Tissue Antigens 12:109–114.

Chan SH, Lin YN, Wee GB, Ren EC, Lui KF, Cheah JS. 1993. Human leucocyte antigen DNA typing in Singaporean Chinese patients with Graves' disease. Ann Acad Med Singapore 22:576–579.

Chen QY, Huang W, She JX, Baxter F, Volpe R, Maclaren NK. 1999. HLA-DRB1*08, DRB1*03/DRB3*0101, and DRB3*0202 are susceptibility genes for Graves' disease in North American Caucasians, whereas DRB1*07 is protective. J Clin Endocrinol Metab 84:3182–3186.

Chen QY, Nadell D, Zhang XY, Kukreja A, Huang YJ, Wise J, Svec F, Richards R, Friday KE, Vargas A, Gomez R, Chalew S, Lan MS, Tomer Y, Maclaren NK. 2000a. The human leukocyte antigen HLA DRB3*020/DQA1*0501 haplotype is associated with Graves' disease in African Americans. J Clin Endocrinol Metab 85:1545–1549.

Chen QY, Nadell D, Zhang XY, Kukreja A, Huang YJ, Wise J, Svec F, Richards R, Friday KE, Vargas A, Gomez R, Chalew S, Lan MS, Tomer Y, Maclaren NK. 2000b. The human leukocyte antigen HLA DRB3*020/DQA1*0501 haplotype is associated with Graves' disease in African Americans. J Clin Endocrinol Metab 85:1545–1549.

Chistiakov DA, Savost'anov KV, Turakulov RI, Petunina N, Balabolkin MI, Nosikov VV. 2002. Further studies of genetic susceptibility to Graves' disease in a Russian population. Med Sci Monit 8:CR180–CR184.

Chistiakov DA, Savost'anov KV, Turakulov RI, Petunina NA, Trukhina LV, Kudinova AV, Balabolkin MI, Nosikov VV. 2000. Complex association analysis of Graves disease using a set of polymorphic markers. Mol Genet Metab 70:214–218.

Cho BY, Rhee BD, Lee DS, Lee MS, Kim GY, Lee HK, Koh CS, Min HK, Lee M. 1987. HLA and Graves' disease in Koreans. Tissue Antigens 30:119–121.

Collins JE, Heward JM, CarrSmith J, Daykin J, Franklyn JA, Gough SC. 2003. Association of a rare thyroglobulin gene microsatellite variant with autoimmune thyroid disease. J Clin Endocrinol Metab 88:5039–5042.

Coyle AJ, Lehar S, Lloyd C, Tian J, Delaney T, Manning S, Nguyen T, Burwell T, Schneider H, Gonzalo JA, Gosselin M, Owen LR, Rudd CE, Gutierrez-Ramos JC. 2000. The CD28-related molecule ICOS is required for effective T cell-dependent immune responses. Immunity 13:95–105.

Cuddihy RM, Bahn RS. 1996. Lack of an association between alleles of interleukin-1 alpha and interleukin-1 receptor antagonsit genes and Graves' disease in a north American Caucasian population. J Clin Endocrinol Metab 81:4476–4478.
Cuddihy RM, Dutton CM, Bahn RS. 1995. A polymorphism in the extracellular domain of the thyrotropin receptor is highly associated with autoimmune thyroid disease in females. Thyroid 5:89–95.
Dahlberg PA, Holmlund G, Karlsson FA, Safwenberg J. 1981. HLA-A, -B, -C and -DR antigens in patients with Graves' disease and their correlation with signs and clinical course. Acta Endocrinol (Copenh) 97:42–47.
Davies TF. 2000. Graves' diseases: pathogenesis. In: Braverman LE, Utiger RD, editors. Werner and Ingbar's The thyroid: A fundamental and clinical text. Philadelphia: Lippincott Williams & Wilkens. p 518–530.
De Roux N, Shields DC, Misrahi M, Ratanachaiyavong S, McGregor AM, Milgrom E. 1996. Analysis of the thyrotropin receptor as a candidate gene in familial Graves' disease. J Clin Endocrinol Metab 81:3483–3486.
Demaine A, Welsh KI, Hawe BS, Farid NR. 1987. Polymorphism of the T cell receptor beta-chain in Graves' disease. J Clin Endocrinol Metab 65:643–646.
Dong RP, Kimura A, Okubo R, Shinagawa H, Tamai H, Nishimura Y, Sasazuki T. 1992. HLA-A and DPB1 loci confer susceptibility to Graves' disease. Hum Immunol 35:165–172.
Donner H, Rau H, Walfish PG, Braun J, Siegmund T, Finke R, Herwig J, Usadel KH, Badenhoop K. 1997a. CTLA4 alanine-17 confers genetic susceptibility to Graves' disease and to type 1 diabetes mellitus. J Clin Endocrinol Metab 82:143–146.
Donner H, Braun J, Seidl C, Rau H, Finke R, Ventz M, Walfish PG, Usadel KH, Badenhoop K. 1997b. Codon 17 polymorphism of the cytotoxic T lymphocyte antigen 4 gene in Hashimoto's thyroiditis and Addison's disease. J Clin Endocrinol Metab 82:4130–4132.
Durie FH, Foy TM, Masters SR, Laman JD, Noelle RJ. 1994. The role of CD40 in the regulation of humoral and cell-mediated immunity. Immunol. Today 15:406–411.
Fakhfakh F, Maalej A, Makni H, Abid M, Jouida J, Zouali M, Ayadi H. 1999. Analysis of immunoglobulin VH and TCR cbeta polymorphisms in a large family with thyroid autoimmune disorder. Exp Clin Immunogenet 16:185–191.
Farid NR. 1981. Graves' disease. In: Farid NR, editor. HLA in endocrine and metabolic disorders. Academic Press. p 85–143.
Farid NR, Sampson L, Noel EP, Barnard JM, Mandeville R, Larsen B, Marshall WH, Carter ND. 1979. A study of human D locus related antigens in Graves' disease. J Clin Investig 63:108–113.
Farid NR, Stone E, Johnson G. 1980. Graves' disease and HLA: Clinical and epidemiologic associations. Clin Endocrinol (Oxf) 13:535–544.
Farid NR, Sampson L, Moens H, Barnard JM. 1981. The association of goitrous autoimmune thyroiditis with HLA-DR5. Tissue Antigens 17:265–268.
Foy TM, Aruffo A, Bajorath J, Buhlmann JE, Noelle RJ. 1996. Immune regulation by CD40 and its ligand GP39. Annu Rev Immunol 14:591–617.
Hall R, Stanbury JB. 1967. Familial studies of autoimmune thyroiditis. Clin Exp Immunol 2:719–725.
Hawkins BR, Ma JT, Lam KS, Wang CC, Yeung RT. 1985a. Analysis of linkage between HLA haplotype and susceptibility to Graves' disease in multiple-case Chinese families in Hong Kong. Acta Endocrinol (Copenh) 110:66–69.
Hawkins BR, Ma JT, Lam KS, Wang CC, Yeung RT. 1985b. Association of HLA antigens with thyrotoxic Graves' disease and periodic paralysis in Hong Kong Chinese. Clin Endocrinol (Oxf) 23:245–252.
Hawkins BR, Lam KSL, Ma JTC, Wang C, Yeung RTT. 1987. Strong association between HLA-DRw9 and Hashimoto's thyroiditis in Southern Chinese. Acta Endocrinol 114:543–546.
Heward JM, Allahabadia A, Daykin J, Carr-Smith J, Daly A, Armitage M, Dodson PM, Sheppard MC, Barnett AH, Franklyn JA, Gough SC. 1998a. Linkage disequilibrium between the human leukocyte antigen class II region of the major histocompatibility complex and Graves' disease: Replication using a population case control and family-based study. J Clin Endocrinol Metab 83:3394–3397.
Heward JM, Allahabadia A, Carr-Smith J, Daykin J, Cockram CS, Gordon CBAH, Franklyn JA, Gough SCL. 1998b. No evidence for allelic association of human CTLA-4 promoter polymorphism with autoimmune thyroid disease in either population-based case-control or family-based studies. Clin Endocrinol 49:331–334.
Heward JM, Allahabadia A, Armitage M, Hattersley A, Dodson PM, Macleod K, Carr-Smith J, Daykin J, Daly A, Sheppard MC, Holder RL, Barnett AH, Franklyn JA, Gough SC. 1999a. The development of Graves' disease and the CTLA-4 gene on chromosome 2q33. J Clin Endocrinol Metab 84:2398–2401.
Heward J, Allahabadia A, Gordon C, Sheppard MC, Barnett AH, Franklyn JA, Gough SC. 1999b. The interleukin-1 receptor antagonist gene shows no allelic association with three autoimmune diseases. Thyroid 9:627–628.
Heward JM, Nithiyananthan R, Allahabadia A, Gibson S, Franklyn JA, Gough SC. 2001. No association of an interleukin 4 gene promoter polymorphism with Graves' disease in the United Kingdom. J Clin Endocrinol Metab 86:3861–3863.
Honda K, Tamai H, Morita T, Kuma K, Nishimura Y, Sasazuki T. 1989. Hashimoto's thyroiditis and HLA in Japanese. J Clin Endocrinol Metab 69:1268–1273.
Huang D, Liu L, Noren K, Xia SQ, Trifunovic J, Pirskanen R, Lefvert AK. 1998. Genetic association of Ctla-4 to myasthenia gravis with thymoma. J Neuroimmunol 88:192–198.
Hutloff A, Dittrich AM, Beier KC, Eljaschewitsch B, Kraft R, Anagnostopoulos I, Kroczek RA. 1999. ICOS is an inducible T-cell co-stimulator structurally and functionally related to CD28. Nature 397:263–266.
Inoue D, Sato K, Enomoto T, Sugawa H, Maeda M, Inoko H, Tsuji K, Mori T, Imura H. 1992. Correlation of HLA types and clinical findings in Japanese patients with hyperthyroid Graves' disease: evidence indicating the existence of four subpopulations. Clin Endocrinol (Oxf) 36:75–82.
Irvine WJ, Gray RS, Morris PJ, Ting A. 1978. HLA in primary atrophic hypothyroidism and Hashimoto goitre. J Clin Lab Immunol 3:193–195.
Kaczur V, Takacs M, Szalai C, Falus A, Nagy Z, Berencsi G, Balazs C. 2000. Analysis of the genetic variability of the 1st (CCC/ACC, P52T) and the 10th exons (bp 1012–1704) of the TSH receptor gene in Graves' disease. Eur J Immunogenet 27:17–23.
Kamizono S, Hiromatsu Y, Seki N, Bednarczuk T, Matsumoto H, Kimura A, Itoh K. 2000. A polymorphism of the 5′ flanking region of tumour necrosis factor alpha gene is associated with thyroid-associated ophthalmopathy in Japanese. Clin Endocrinol (Oxf) 52:759–764.
Katsuren E, Awata T, Matsumoto C, Yamamoto K. 1994. HLA class II alleles in Japanese patients with Graves' disease: Weak associations of HLA-DR and -DQ. Endocr J 41:599–603.
Kawa A, Nakamura S, Nakazawa M, Sakaguch S, Kawabata T, Maeda Y, Kanehisa T. 1977. HLA-BW35 and B5 in Japanese patients with Graves' disease. Acta Endocrinol (Copenh) 86:754–757.
Kendall-Taylor P, Stephenson A, Stratton A, Papiha SS, Perros P, Roberts DF. 1988. Differentiation of autoimmune ophthalmopathy from Graves' hyperthyroidism by analysis of genetic markers. Clin Endocrinol (Oxf) 28:601–610.
Kim TY, Park YJ, Hwang JK, Song JY, Park KS, Cho BY, Park DJ. 2003. A C/T polymorphism in the 5′-untranslated region of the CD40 gene is associated with Graves' disease in Koreans. Thyroid 13:919–925.
Kinjo Y, Takasu N, Komiya I, Tomoyose T, Takara M, Kouki T, Shimajiri Y, Yabiku K, Yoshimura H. 2002. Remission of

Graves' hyperthyroidism and A/G polymorphism at position 49 in exon 1 of cytotoxic T lymphocyte-associated molecule-4 gene. J Clin Endocrinol Metab 87:2593–2596.

Kong YC, Lomo LC, Motte RW, Giraldo AA, Baisch J, Strauss G, Hammerling GJ, David CS. 1996. HLA-DRB1 polymorphism determines susceptibility to autoimmune thyroiditis in transgenic mice: Definitive association with HLA- DRB1*0301 (DR3) gene. J Exp Med 184:1167–1172.

Kotsa K, Watson PF, Weetman AP. 1997a. A CTLA-4 gene polymorphism is associated with both Graves' disease and autoimmune hypothyroidism. Clin Endocrinol 46:551–554.

Kotsa KD, Watson PF, Weetman AP. 1997b. No association between a thyrotropin receptor gene polymorphism and Graves' disease in the female population. Thyroid 7:31–33.

Kouki T, Sawai Y, Gardine CA, Fisfalen ME, Alegre ML, DeGroot LJ. 2000. CTLA-4 gene polymorphism at position 49 in exon 1 reduces the inhibitory function of CTLA-4 and contributes to the pathogenesis of Graves' disease. J Immunol 165:6606–6611.

Kouki T, Gardine CA, Yanagawa T, DeGroot LJ. 2002. Relation of three polymorphisms of the CTLA-4 gene in patients with Graves' disease. J Endocrinol Invest 25:208–213.

Maciel LM, Rodrigues SS, Dibbern RS, Navarro PA, Donadi EA. 2001. Association of the HLA-DRB1*0301 and HLA-DQA1*0501 alleles with Graves' disease in a population representing the gene contribution from several ethnic backgrounds. Thyroid 11:31–35.

Mangklabruks A, Cox N, DeGroot LJ. 1991. Genetic factors in autoimmune thyroid disease analyzed by restriction fragment length polymorphisms of candidate genes. J Clin Endocrinol Metab 73:236–244.

Marga M, Denisova A, Sochnev A, Pirags V, Farid NR. 2001. Two HLA DRB 1 alleles confer independent genetic susceptibility to Graves disease: Relevance of cross-population studies. Am J Med Genet 102:188–191.

Marron MP, Raffel LJ, Garchon HJ, Jacob CO, SerranoRios M, Martinez Larrad MT, Teng WP, Park Y, Zhang ZX, Goldstein DR, Tao YW, Beaurain G, Bach JF, Huang HS, Luo DF, Zeidler A, Rotter JI, Yang MCK, Modilevsky T, Maclaren NK, She JX. 1997. Insulin-dependent diabetes mellitus (IDDM) is associated with CTLA4 polymorphisms in multiple ethnic groups. Hum Mol Genet 6:1275–1282.

Marron MP, Zeidler A, Raffel LJ, Eckenrode SE, Yang JJ, Hopkins DI, Garchon HJ, Jacob CO, Serrano-Rios M, Martinez Larrad MT, Park Y, Bach JF, Rotter JI, Yang MC, She JX. 2000. Genetic and physical mapping of a type 1 diabetes susceptibility gene (IDDM12) to a 100-kb phagemid artificial chromosome clone containing D2S72-CTLA4-D2S105 on chromosome 2q33. Diabetes 49:492–499.

Martin L. 1945. The heredity and familial aspects of exophathalmic goitre and nodular goitre. Q. J Med 14:207–219.

Moens H, Farid NR, Sampson L, Noel EP, Barnard JM. 1978. Hashimoto's thyroiditis is associated with HLA-DRw3. N Engl J Med 299:133–134.

Muhlberg T, Kirchberger M, Spitzweg C, Herrmann F, Heberling HJ, Heufelder AE. 1998. Lack of association of Graves' disease with the A2 allele of the interleukin-1 receptor antagonist gene in a white European population. Eur J Endocrinol 138:686–690.

Muhlberg T, Herrmann K, Joba W, Kirchberger M, Heberling HJ, Heufelder AE. 2000. Lack of association of nonautoimmune hyperfunctioning thyroid disorders and a germline polymorphism of codon 727 of the human thyrotropin receptor in a European Caucasian population. J Clin Endocrinol Metab 85:2640–2643.

Nagataki S. 1986. The interaction of MHC and Gm in liability to autoimmune thyroid disease. Mol Biol Med 3:73–84.

Nakao Y, Matsumoto H, Miyazaki T, Nishitani H, Takatsuki K, Kasukawa R, Nakayama S, Izumi S, Fujita T, Tsuji K. 1980. IgG heavy chain allotypes (Gm) in atrophic and goitrous thyroiditis. Clin Exp Immunol 42:20–26.

Nistico L, Buzzetti R, Pritchard LE, Van der Auwera B, Giovannini C, Bosi E, Larrad MT, Rios MS, Chow CC, Cockram CS, Jacobs K, Mijovic C, Bain SC, Barnett AH, Vandewalle CL, Schuit F, Gorus FK, Tosi R, Pozzilli P, Todd JA. 1996. The CTLA-4 gene region of chromosome 2q33 is linked to, and associated with type 1 diabetes. The Belgian Diabetes Registry. Hum Mol Genet 5:1075–1080.

Nithiyananthan R, Heward JM, Allahabadia A, Franklyn JA, Gough SC. 2002. Polymorphism of the CTLA-4 gene is associated with autoimmune hypothyroidism in the United Kingdom. Thyroid 12:3–6.

Ohtsuka K, Nakamura Y. 1998. Human leukocyte antigens associated with hyperthyroid Graves ophthalmology in Japanese patients. Am J Ophthalmol 126:805–810.

Omar MA, Hammond MG, Desai RK, Motala AA, Aboo N, Seedat MA. 1990. HLA class I and II antigens in South African blacks with Graves' disease. Clin Immunol Immunopathol 54:98–102.

Onuma H, Ota M, Sugenoya A, Inoko H. 1994. Association of HLA-DPB1*0501 with early-onset Graves' disease in Japanese. Hum Immunol 39:195–201.

Pani MA, Regulla K, Segni M, Hofmann S, Hufner M, Pasquino AM, Usadel KH, Badenhoop K. 2002. A polymorphism within the vitamin D-binding protein gene is associated with Graves' disease but not with Hashimoto's thyroiditis. J Clin Endocrinol Metab 87:2564–2567.

Park YJ, Chung HK, Park DJ, Kim WB, Kim SW, Koh JJ, Cho BY. 2000. Polymorphism in the promoter and exon 1 of the cytotoxic T lymphocyte antigen-4 gene associated with autoimmune thyroid disease in Koreans. Thyroid 10:453–459.

Pearce SH, Vaidya B, Imrie H, Perros P, Kelly WF, Toft AD, McCarthy MI, Young ET, Kendall-Taylor P. 1999. Further evidence for a susceptibility locus on chromosome 20q13.11 in families with dominant transmission of Graves disease [letter]. Am J Hum Genet 65:1462–1465.

Petrone A, Giorgi G, Mesturino CA, Capizzi M, Cascino I, Nistico L, Osborn J, Di Mario U, Buzzetti R. 2001. Association of DRB1*04-DQB1*0301 haplotype and lack of association of two polymorphic sites at CTLA-4 gene with Hashimoto's thyroiditis in an Italian population. Thyroid 11:171–175.

Phillips DI, Osmond C, Baird J, Huckle A, Rees-Smith B. 2002. Is birthweight associated with thyroid autoimmunity? A study in twins. Thyroid 12:377–380.

Pirro MT, De Filippis V, Di Cerbo A, Scillitani A, Liuzzi A, Tassi V. 1995. Thyroperoxidase microsatellite polymorphism in thyroid disease. Thyroid 5:461–464.

Rapoport B, Chazenbalk GD, Jaume JC, McLachlan SM. 1998. The thyrotropin (TSH) receptor: Interaction with TSH and autoantibodies. Endocr Rev 19:673–716.

Rau H, Nicolay A, Usadel KH, Finke R, Donner H, Walfish PG, Badenhoop K. 1997. Polymorphisms of TAP1 and TAP2 genes in Graves' disease. Tissue Antigens 49:16–22.

Reiser H, Stadecker MJ. 1996. Costimulatory B7 molecules in the pathogenesis of infectious and autoimmune diseases. N Engl J Med 335:1369–1377.

Ringold DA, Nicoloff JT, Kesler M, Davis H, Hamilton A, Mack T. 2002. Further evidence for a strong genetic influence on the development of autoimmune thyroid disease: The California twin study. Thyroid 12:647–653.

Risch N. 1990. Linkage strategies for genetically complex traits. II. The power of affected relative pairs. Am J Hum Genet 46:229–241.

Roman, S.H., Hubbard, M. and Rubinstein, P. (1989) Failure to confirm standard HLA and Gm immunogenetic typing as a predictor of familial autoimmune thyroid disease. The 74th Annual Meeting of the Endocrine Society, Seattle, WA June.

Roman SH, Greenberg DA, Rubinstein P, Wallenstein S, Davies TF. 1992. Genetics of autoimmune thyroid disease: Lack of evidence for linkage to HLA within families. J Clin Endocrinol Metab 74:496–503.

Sakai K, Shirasawa S, Ishikawa N, Ito K, Tamai H, Kuma K, Akamizu T, Tanimura M, Furugaki K, Yamamoto K, Sasazuki T. 2001. Identification of susceptibility loci for autoimmune thyroid disease to 5q31-q33 and Hashimoto's thyroiditis to 8q23-q24 by multipoint affected sib-pair linkage analysis in Japanese. Hum Mol Genet 10:1379–1386.

Sale MM, Akamizu T, Howard TD, Yokota T, Nakao K, Mori T, Iwasaki H, Rich SS, Jennings-Gee JE, Yamada M, Bowden DW. 1997. Association of autoimmune thyroid disease with a microsatellite marker for the thyrotropin receptor gene and CTLA-4 in a Japanese population. Proc Assoc Am Physicians 109:453–461.

Sawai Y, DeGroot LJ. 2000. Binding of human thyrotropin receptor peptides to a Graves' disease- predisposing human leukocyte antigen class II molecule. J Clin Endocrinol Metab 85:1176–1179.

Schleusener H, Schwander J, Fischer C, Holle R, Holl G, Badenhoop K, Hensen J, Finke R, Bogner U, Mayr WR. 1989. Prospective multicentre study on the prediction of relapse after antithyroid drug treatment in patients with Graves' disease. Acta Endocrinol (Copenh) 120:689–701.

Segni M, Pani MA, Pasquino AM, Badenhoop K. 2002. Familial clustering of juvenile thyroid autoimmunity: Higher risk is conferred by human leukocyte antigen DR3-DQ2 and thyroid peroxidase antibody status in fathers. J Clin Endocrinol Metab 87:3779–3782.

Shields DC, Ratanachaiyavong S, McGregor AM, Collins A, Morton NE. 1994. Combined segregation and linkage analysis of Graves' disease with a thyroid autoantibody diathesis. Am J Hum Genet 55:540–554.

Siegmund T, Usadel KH, Donner H, Braun J, Walfish PG, Badenhoop K. 1998. Interferon-gamma gene microsatellite polymorphisms in patients with Graves' disease. Thyroid 8:1013–1017.

Simanainen J, Kinch A, Westermark K, Winsa B, Bengtsson M, Schuppert F, Westermark B, Heldin NE. 1999. Analysis of mutations in exon 1 of the human thyrotropin receptor gene: High frequency of the D36H and P52T polymorphic variants. Thyroid 9:7–11.

Sridama V, Hara Y, Fauchet R, DeGroot LJ. 1987. HLA immunogenetic heterogenity in Black American patients with Graves' disease. Arch Intern Med 147:229–231.

Stenszky V, Kozma L, Balazs C, Rochkitz S, Bear JC, Farid NR. 1985. The genetics of Graves' disease: HLA and disease susceptibility. J Clin Endocrinol Metab 61:735–740.

Tan S, Chan S, Lee B, Wee G, Wong H. 1988. HLA association in Singapore children with Grave's disease. Metabolism 37:518–519.

Tandon N, Mehra NK, Taneja V, Vaidya MC, Kochupillai N. 1990. HLA antigens in Asian Indian patients with Graves' disease. Clin Endocrinol (Oxf) 33:21–26.

Tandon N, Zhang L, Weetman AP. 1991. HLA associations with Hashimoto's thyroiditis. Clin Endocrinol (Oxf) 34:383–386.

Todd JA, Bell JI, McDevitt HO. 1987. HLA-DQ beta gene contributes to susceptibility and resistance to insulin-dependent diabetes mellitus. Nature 329:599–604.

Todd JA, Acha-Orbea H, Bell JI, Chao N, Fronek Z, Jacob CO, McDermott M, Sinha AA, Timmerman L, Steinman L, et al. 1988. A molecular basis for MHC class II–associated autoimmunity. Science 240:1003–1009.

Tomer Y. 2001. Unraveling the genetic susceptibility to autoimmune thyroid diseases: CTLA-4 takes the stage. Thyroid 11:167–169.

Tomer Y, Barbesino G, Greenberg DA, Davies TF. 1997a. The immunogenetics of autoimmune diabetes and autoimmune thyroid disease. Trends Endocrinol Metab 8:63–70.

Tomer Y, Barbesino G, Keddache M, Greenberg DA, Davies TF. 1997b. Mapping of a major susceptibility locus for Graves' disease (GD-1) to chromosome 14q31. J Clin Endocrinol Metab 82:1645–1648.

Tomer Y, Barbesino G, Greenberg DA, Concepcion ES, Davies TF. 1998. A new Graves disease-susceptibility locus maps to chromosome 20q11.2. Am J Hum Genet 63:1749–1756.

Tomer Y, Barbesino G, Greenberg DA, Concepcion ES, Davies TF. 1999. Mapping the major susceptibility loci for familial Graves' and Hashimoto's diseases: Evidence for genetic heterogeneity and gene interactions. J Clin Endocrinol Metab 84:4656–4664.

Tomer Y, Greenberg DA, Barbesino G, Concepcion ES, Davies TF. 2001. CTLA-4 and not CD28 is a susceptibility gene for thyroid autoantibody production. J Clin Endocrinol Metab 86:1687–1693.

Tomer Y, Concepcion E, Greenberg DA. 2002a. A C/T single nucleotide polymorphism in the region of the CD40 gene is associated with Graves' disease. Thyroid 12:1129–1135.

Tomer Y, Greenberg DA, Concepcion E, Ban Y, Davies TF. 2002b. Thyroglobulin is a thyroid specific gene for the familial autoimmune thyroid diseases. J Clin Endocrinol Metab 87:404–407.

Tonacchera M, Pinchera A. 2000. Thyrotropin receptor polymorphisms and thyroid diseases. J Clin Endocrinol Metab 85:2637–2639.

Ueda H, Howson JM, Esposito L, Heward J, Snook H, Chamberlain G, Rainbow DB, Hunter KM, Smith AN, Di Genova G, Herr MH, Dahlman I, Payne F, Smyth D, Lowe C, Twells RC, Howlett S, Healy B, Nutland S, Rance HE, Everett V, Smink LJ, Lam AC, Cordell HJ, Walker NM, Bordin C, Hulme J, Motzo C, Cucca F, Hess JF, Metzker ML, Rogers J, Gregory S, Allahabadia A, Nithiyananthan R, Tuomilehto-Wolf E, Tuomilehto J, Bingley P, Gillespie KM, Undlien DE, Ronningen KS, Guja C, Ionescu-Tirgoviste C, Savage DA, Maxwell AP, Carson DJ, Patterson CC, Franklyn JA, Clayton DG, Peterson LB, Wicker LS, Todd JA, Gough SC. 2003. Association of the T-cell regulatory gene CTLA4 with susceptibility to autoimmune disease. Nature 423:506–511.

Vaidya B, Imrie H, Perros P, Young ET, Kelly WF, Carr D, Large DM, Toft AD, McCarthy MI, Kendall-Taylor P, Pearce SH. 1999a. The cytotoxic T lymphocyte antigen-4 is a major Graves' disease locus. Hum Mol Genet 8: 1195–1199.

Vaidya B, Imrie H, Perros P, Dickinson J, McCarthy MI, Kendall-Taylor P, Pearce SH. 1999b. Cytotoxic T lymphocyte antigen-4 (CTLA-4) gene polymorphism confers susceptibility to thyroid associated orbitopathy [letter]. Lancet 354:743–744.

Vaidya B, Imrie H, Geatch DR, Perros P, Ball SG, Baylis PH, Carr D, Hurel SJ, James RA, Kelly WF, Kemp EH, Young ET, Weetman AP, Kendall-Taylor P, Pearce SH. 2000. Association analysis of the cytotoxic T lymphocyte antigen-4 (CTLA-4) and autoimmune regulator-1 (AIRE-1) genes in sporadic autoimmune Addison's disease. J Clin Endocrinol Metab 85:688–691.

Vanderpump MPJ, Tunbridge WMG, French JM, Appleton D, Bates D, Clark F, Grimley Evans J, Hasan DM, Rodgers H, Tunbridge F, Young ET. 1995. The incidence of thyroid disorders in the community: A twenty-year follow-up of the Whickham survey. Clin Endocrinol (Oxf) 43:55–68.

Villanueva RB, Inzerillo AM, Tomer Y, Barbesino G, Meltzer M, Concepcion ES, Greenberg DA, Maclaren N, Sun ZS, Zhang DM, Tucci S, Davies TF. 2000. Limited genetic susceptibility to severe Graves' ophthalmopathy: No role for ctla-4 and evidence for an environmental etiology. Thyroid 10:791–798.

Volpe R. 1990. Immunology of human thyroid disease. In: Volpe R, editor. Autoimmunity in Endocrine Disease. Boca Raton: CRC Press. p 73.

Vyse TJ, Todd JA. 1996. Genetic analysis of autoimmune disease. Cell 85:311–318.

Weetman AP. 1996. Chronic autoimmune thyroiditis. In: Braverman LE, Utiger RD, editors. Werner and Ingbar's The Thyroid. Philadelphia: Lippincott-Raven. p 738–748.

Weetman AP, So AK, Roe C, Walport MJ, Foroni L. 1987. T-cell receptor alpha chain V region polymorphism linked to primary autoimmune hypothyroidism but not Graves' disease. Hum Immunol 20:167–173.

Weetman AP, So AK, Warner CA, Foroni L, Fells P, Shine B. 1988. Immunogenetics of Graves' ophthalmopathy. Clin Endocrinol 28:619–628.

Wood JP, Pani MA, Bieda K, Meyer G, Usadel KH, Badenhoop K. 2002. A recently described polymorphism in the CD28 gene on chromosome 2q33 is not associated with susceptibility to type 1 diabetes. Eur J Immunogenet 29:347–349.

Wu Z, Stephens HAF, Sachs JA, Biro PA, Cutbush S, Magzoub MM, Becker C, Schwartz G, Botazzo GF. 1994. Molecular analysis of HLA-DQ and -DP genes in caucasoid patients with Hashimoto's thyroiditis. Tissue Antigens 43:116–119.

Xu Y, Graves P, Tomer Y, Davies T. 2002. CTLA-4 and autoimmune thyroid disease: lack of influence of the A49G signal peptide polymorphism on functional recombinant human CTLA-4. Cell Immunol 215:133.

Yamazaki K, Takazoe M, Tanaka T, Kazumori T, Nakamura Y. 2002. Absence of mutation in the NOD2/CARD15 gene among 483 Japanese patients with Crohn's disease. J Hum Genet 47:469–472.

Yanagawa T, Mangklabruks A, Chang YB, Okamoto Y, Fisfalen M-E, Curran PG, DeGroot LJ. 1993. Human histocompatibility leukocyte antigen-DQA1*0501 allele associated with genetic susceptibility to Graves' disease in a Caucasian population. J Clin Endocrinol Metab 76:1569–1574.

Yanagawa T, Hidaka Y, Guimaraes V, Soliman M, DeGroot LJ. 1995. CTLA-4 gene polymorphism associated with Graves' disease in a Caucasian population. J Clin Endocrinol Metab 80:41–45.

Yanagawa T, Taniyama M, Enomoto S, Gomi K, Maruyama H, Ban Y, Saruta T. 1997. CTLA4 gene polymorphism confers susceptibility to Graves' disease in Japanese. Thyroid 7:843–846.

Yeo PP, Chan SH, Thai AC, Ng WY, Lui KF, Wee GB, Tan SH, Lee BW, Wong HB, Cheah JS. 1989. HLA Bw46 and DR9 associations in Graves' disease of Chinese patients are age- and sex-related. Tissue Antigens 34:179–184.

Zaletel K, Krhin B, Gaberscek S, Pirnat E, Hojker S. 2002. The influence of the exon 1 polymorphism of the cytotoxic T lymphocyte antigen 4 gene on thyroid antibody production in patients with newly diagnosed Graves' disease. Thyroid 12:373–376.

Zamani M, Spaepen M, Bex M, Bouillon R, Cassiman JJ. 2000. Primary role of the HLA class II DRB1*0301 allele in Graves disease. Am J Med Genet 95:432–437.

